# Can Adjunctive Lithium Therapy Influence Emotional Dysregulation in Adolescents? Findings from a Retrospective Study

**DOI:** 10.3390/jcm14134807

**Published:** 2025-07-07

**Authors:** Federica Gigliotti, Luca Cammisa, Sara Riezzo, Arianna Terrinoni

**Affiliations:** 1 Department of Human Neuroscience, Sapienza University of Rome, 00185 Rome, Italy; federica.gig@uniroma1.it (F.G.); riezzosara@gmail.com (S.R.); 2 Behavioural Neuroscience, Department of Psychology, Sapienza University of Rome, 00185 Rome, Italy; luca.cammisa@uniroma1.it

**Keywords:** lithium, adolescents, emotional dysregulation

## Abstract

**Background**: Emotional dysregulation (ED) is a transdiagnostic feature of multiple adolescent psychiatric disorders and a predictor of functional impairment and self-harming behaviors. Despite its clinical relevance, pharmacological treatments targeting ED in youth remain underexplored. This retrospective study investigated the clinical effectiveness and tolerability of adjunctive lithium therapy in adolescents with severe ED, independent of specific diagnostic categories. **Methods**: A total of 35 inpatients (13–17 years) with significant ED were divided into two groups based on pharmacological treatment: lithium add-on therapy (Li group, *n* = 17) and standard therapy without lithium (Control group, *n* = 18). Clinical severity (CGI-S) and global functioning (C-GAS) were assessed at baseline (T0), 6 months (T1), and 12 months (T2). A mixed-design ANOVA was performed to assess group × time interactions. Adverse events and treatment adherence were also examined. **Results**: At T1, the Li group showed a significantly greater reduction in symptom severity (CGI-S) compared to the Control group (*p* = 0.029). Global functioning (C-GAS) improved over time in both groups (*p* < 0.001), with no significant interaction effects. Adverse effects, primarily metabolic and endocrine, were more frequent in the Li group but did not reduce adherence. **Conclusions**: Adjunctive lithium therapy may reduce symptom severity in adolescents with severe ED without negatively affecting treatment tolerability or adherence. These findings support the potential utility of lithium in complex adolescent cases and warrant further prospective research.

## 1. Introduction

Emotional dysregulation (ED) is increasingly regarded as a core transdiagnostic dimension underlying a wide spectrum of childhood and adolescent psychopathology, ranging from mood and anxiety disturbances to behavioral and personality disorders [1,2]. Broadly defined as difficulty in monitoring, evaluating, and modulating emotional responses in accordance with internal goals or external demands, ED involves deficits in both awareness and control of affective states [3], often resulting in significant functional impairment and heightened risk for comorbidity [4]. Conceptually, this construct reflects the interplay among temperament, neurocognitive development, and environmental experiences, positioning ED at the intersection of affective neuroscience and developmental psychopathology.

During adolescence—a critical developmental window—the balance between emotional and regulatory brain systems is still maturing. Neurodevelopmental evidence has consistently shown that limbic and paralimbic structures (e.g., amygdala, striatum) mature earlier than the prefrontal cortex, which is responsible for top-down emotional regulation, impulse control, and social cognition [5,6]. This maturational gap contributes to increased affective instability and impulsive behaviors, as well as greater vulnerability to a range of adolescent-onset conditions (e.g., bipolar disorder, borderline personality disorder, and conduct disorders) [2,7,8], particularly when exacerbated by environmental stressors or genetic predispositions [9].

In clinical populations, ED transcends traditional diagnostic boundaries. It is a core feature of disorders such as disruptive mood dysregulation disorder (DMDD), borderline personality disorder (BPD), bipolar disorder (BD), oppositional defiant disorder (ODD), and conduct disorder (CD) [10,11]. Additionally, ED is a predictive factor for self-harm, suicidality, aggression, and long-term functional impairment [12,13].

Despite its transdiagnostic nature and high clinical relevance, pharmacological strategies specifically targeting ED in adolescents are often extrapolated from adult studies, with limited empirical validation in pediatric populations. Selective serotonin reuptake inhibitors (SSRIs), second-generation antipsychotics, and mood stabilizers are commonly prescribed; however, their effectiveness on core dysregulatory symptoms remains inconclusive [4,14].

In this context, lithium—one of the oldest and most extensively studied mood stabilizers—has re-emerged as a potentially valuable agent for adolescents with ED. Originally approved for acute mania and maintenance treatment of bipolar disorder in adults, lithium exerts wide-ranging neurobiological effects, including modulation of serotonergic and glutamatergic neurotransmission, inhibition of inositol monophosphatase, attenuation of glycogen synthase kinase-3β (GSK3β) activity, and up-regulation of neurotrophic factors such as BDNF [15,16]. Notably, lithium has demonstrated anti-impulsive, anti-aggressive, and anti-suicidal properties, particularly relevant for populations characterized by marked emotional lability and deficits in behavioral control [17,18].

Although traditionally used for treating bipolar disorder, recent findings support the use of lithium in adolescents with severe mood and behavioral dysregulation, particularly when aggressive outbursts, irritability, or emotional lability are prominent [19,20]. Emerging studies suggest that lithium’s anti-impulsive and anti-aggressive effects may result from its serotonergic modulation, GSK3 β inhibition, and enhancement of neurotrophic signaling [21]. Furthermore, a growing body of literature supports lithium’s unique ability to reduce the risk of suicidal behavior, not only in bipolar populations but also in broader mood and behavioral disorders [22,23].

Nevertheless, clinical use of lithium in adolescents remains controversial, primarily due to concerns regarding its restricted therapeutic window, potential adverse effects (e.g., renal and thyroid dysfunction), and the need for regular serum monitoring [24,25]. Moreover, most of the existing evidence derives from small-scale trials focused on limited diagnostic populations, reducing the generalizability of findings [26,27]. These challenges, combined with the paucity of long-term pediatric studies and the preference for newer psychotropic medications, have contributed to lithium being relegated to a secondary role in many treatment protocols—despite growing evidence of its utility.

In light of these findings, the present retrospective study aims to investigate the clinical efficacy and tolerability of adjunctive lithium therapy in adolescents with significant emotional dysregulation, regardless of primary diagnosis. By evaluating both symptomatic outcomes and tolerability profiles in an inpatient cohort, this study seeks to contribute to the growing body of evidence on the potential role of lithium as a therapeutic tool for severe emotional and behavioral dysregulation in adolescence.

## 2. Materials and Methods

### 2.1. Participants and Procedure

A total of thirty-five adolescents with a history of ED, ranging from 13 to 17 years of age, were included in the present study. Data were collected from medical records of inpatient adolescents referred to our third-level University Hospital between September 2022 and September 2024. Selection criteria included the presence of clinically significant emotional dysregulation, as measured by Difficulties in Emotion Regulation Scale (DERS) [28], and an age range between 13 and 18 years. Exclusion criteria were represented by the ascertained presence of associated neurological conditions (e.g., epilepsy), and a previous or current diagnosis of autism spectrum disorder, intellectual disability, or schizophrenia spectrum/other psychotic disorders according to DSM-5-TR [10].

The total sample was divided into two subgroups depending on whether adjunctive lithium therapy was administered as part of the pharmacological treatment (Li group) or not (Control group). All participants were monitored for a minimum period of 6 months (T1); among these, an extended follow-up period of 12 months (T2) was available for 27 patients.

Written informed consent was obtained from all patients’ parents or legal guardians at the time of admission, authorizing the inclusion of clinical data in research analysis and the anonymous reporting of aggregate findings, in compliance with Italian legal and ethical standards. As all assessments and procedures analyzed were part of standard clinical care routinely implemented at our center, ethical approval was not required. The study was conducted in accordance with the principles of the Declaration of Helsinki.

For all patients included in the study, medical records were examined to collect both anamnestic and clinical data. The baseline time point (T0) was defined as the time of initial clinical assessment at the admission for the Control group, and to the introduction of lithium treatment during hospitalization for the Li group.

At T0, the following anamnestic variables were recorded: gender, age, family history of psychiatric disorders, age at clinical onset, previous suicidal attempts/self-injurious behaviors, and any previous treatments. Clinical data collected at T0 included scores on the DERS [28], primary psychiatric diagnosis, personality organization according to DSM-5-TR criteria [10], drug therapy, severity of illness (measured using the Clinical Global Impression–Severity scale, CGI-S) [29], and level of global functioning (assessed via the Children’s Global Assessment Scale, C-GAS) [30].

Follow-up clinical data were extracted from medical records at 6 months (T1) and 12 months (T2). These included illness severity (CGI-S), global functioning (C-GAS), current pharmacological treatment, number of psychiatric rehospitalizations, significant adverse events, and treatment adherence. For the subjects in the Li group, the serum lithium levels were also recorded.

### 2.2. Measures

ED was assessed using the DERS [28], a 36-item self-report questionnaire widely recognized as one of the most comprehensive psychometric instruments in this field [31]. This instrument evaluates individual characteristic patterns of emotion regulation across six distinct dimensions: (a) lack of awareness of emotional responses (Awareness), reflecting difficulties and/or unwillingness to attend to and acknowledge emotions; (b) lack of clarity of emotional responses (Clarity), describing the difficulties in identifying and understanding one’s emotional state; (c) non-acceptance of emotional responses (Non-acceptance), reflecting the tendency to experience secondary negative reactions (e.g., shame, guilt) in response to one’s own negative emotions; (d) limited access to emotional regulation strategies perceived as effective (Strategies), describing the perceived inability to effectively regulate emotions once activated; (e) difficulties in controlling impulses when experiencing negative emotions (Impulse), assessing the difficulties to remain behaviorally controlled when experiencing intense negative emotions; (f) difficulties in engaging in goal-directed behaviors when experiencing negative emotions (Goals), measuring the extent to which negative emotions interfere with concentration and task completion. Each item is rated on a 5-point Likert scale (1 = “almost never” to 5 = “almost always”), with the total score ranging from 36 to 180; higher scores indicating greater difficulties in emotion regulation. The DERS has shown good psychometric properties, including excellent internal consistency for the total scale (Cronbach’s α = 0.93) and good reliability across subscales (Cronbach’s α ranging from 0.80 to 0.89) [28]. Patients included in the present study presented clinically significant scores on at least three of the six DERS subscales.

Illness severity at baseline and during follow-up was assessed using the CGI-S, a single-item measure that requires the clinician to rate the severity of global symptomatology at the time of assessment on a scale ranging from 1 (“normal, not at all ill”) to 7 (“among the most extremely ill patients”). Ratings are based on the clinician’s overall impression, integrating all available information, including clinical interviews, behavioral observations, and collateral reports [29].

Global functioning was assessed using the C-GAS, a clinician-rated instrument designed to evaluate the overall level of psychological, social, and occupational functioning in children and adolescents aged 4 to 18 years. The C-GAS provides a single score ranging from 1 (severely impaired) to 100 (superior functioning), with higher scores indicating better adaptive functioning. Ratings are based on clinical judgment integrating information from multiple sources (e.g., interviews, observations, reports) and refer to the child’s functioning, excluding the influence of physical or environmental limitations. The scale is divided into ten-point intervals, each corresponding to descriptive anchors outlining the degree of impairment [30].

Adverse effects were systematically monitored through both clinical observation and review of medical records at each follow-up visit (baseline, 6 months, and 12 months). Monitoring included not only serum lithium levels but also renal (serum creatinine, BUN), thyroid (TSH, free T4), metabolic (fasting glucose, lipids), and cardiac (EKG) parameters. Adverse events were categorized based on their presence and frequency, rather than on severity grading. Specifically, patients were classified into three categories: no reported adverse effects, one adverse effect, or more than one adverse effect.

### 2.3. Statistical Analysis

All data analyses were conducted using IBM Statistical Package for Social Science for Windows (SPSS), version 25.0. Descriptive statistics were used to analyze sociodemographic and clinical characteristics. Continuous data were reported as means ± standard deviations (SDs), while categorical variables were presented as frequencies and percentages. Group differences in continuous variables were examined using one-way analysis of variance (ANOVA), whereas categorical variables were compared using chi-square (χ^2^) tests. To evaluate the clinical efficacy of adjunctive lithium therapy, a mixed-design ANOVA was performed to compare improvements in both illness severity (CGI-S) and level of global functioning (C-GAS) over time between the two groups. Statistical significance was set at *p* ≤ 0.05.

## 3. Results

### 3.1. Baseline

A total of 35 patients were included into our study, comprising 31 females (88.6%) and 4 males (11.4%), with a mean age of 15.23 years (SD = 1.61). Of these, 17 patients (48.6%) were allocated in the Li group and 18 (51.4%) in the Control group. Table 1 reports a comparison of demographic and clinical data between the two groups. Some statistically significant differences were observed in some baseline variables; specifically, the Li group exhibited a significantly higher number of previous psychiatric hospitalizations (F(1,33) = 27.628, *p* < 0.001, η^2^ = 0.456). Moreover, all patients in the Li group (100%) had a history of prior pharmacological treatment, compared to only 22.2% in the Control group (χ^2^(1, *n* = 35) = 22.037, *p* < 0.001). The effect size, as measured by Cramér’s V, was 0.793, indicating a large association. Finally, the mean number of prescribed psychotropic medications was significantly higher in the Li group than in the Control group (F = 21.270, *p* < 0.001, η^2^ = 0.392).

DERS subscales analysis revealed a significant between-group difference only for the Awareness dimension, with higher scores observed in the Li group (F(1,33) = 4.508, *p* = 0.041, η^2^ = 0.12) (see Table 2).

According to DSM-5-TR criteria [10], primary psychiatric diagnoses within the total sample were grouped into four categories: bipolar spectrum disorders (17%); depressive disorders (43%); disruptive, impulse-control, and conduct disorders (11%); and comorbid presentations of mood and disruptive disorders (29%). Personality traits were categorized according to the DSM-5-TR cluster classification [10]. Borderline personality organization was considered independently due to its high prevalence in the total sample (54%). Among the remaining patients, 17% met criteria for a cluster B organization (excluding borderline), 14% for cluster C, and 3% for cluster A. No statistically significant differences were observed between the two subgroups in the distribution of primary psychiatric diagnoses (χ^2^ (3, *n* = 35) = 3.241, *p* = 0.3559) and personality profile (χ^2^ (4, *n* = 35) = 6.496, *p* = 0.165).

Regarding the pharmacological treatment profile, in the Control group, second-generation antipsychotics (50%) and serotonin reuptake inhibitors (44.4%) were the most frequently prescribed, followed by antiepileptics (22.2%). In contrast, the Li group showed a predominance of antipsychotic use (76.5%), with lower rates of serotonergic (17.6%) and antiepileptic (5.6%) prescriptions.

### 3.2. Follow-Up

As reported in Table 3, analysis of secondary outcomes at T1 revealed no significant differences between the two groups, except for the mean number of prescribed medications, which was significantly higher in the Li group (F(1,33) = 11.760, *p* = 0.002, η^2^ = 0.261). At T2, the Li group exhibited a significantly greater number of psychiatric hospitalizations compared to the Control group (F(1,25) = 5.829, *p* = 0.023, η^2^ = 0.189), while no other secondary outcomes showed statistically significant differences.

The distribution of medication classes remained largely stable at both T1 and T2, showing proportions similar to those observed at baseline.

Serum lithium levels in the Li group averaged 0.75 mEq/L (SD = 0.17) at T1 and 0.72 mEq/L (SD = 0.17) at T2.

As shown in Table 4, a mixed-model ANOVA was conducted to compare CGI-S and C-GAS scores across three time points. Prior to the analysis, the assumption of normality was assessed, revealing non-normal distributions of CGI scores at T0, T1, and T2. Visual inspection of histograms and Q–Q plots suggested moderately skewed distributions. This was confirmed by the skewness values observed at each time point (T0 = −0.79; T1 = 0.90; T2 = −0.76), indicating a moderate departure from symmetry. Despite these deviations, mixed-model ANOVA was appropriate, as the procedure is considered robust to moderate violations of normality, particularly in studies with balanced group sizes and sample sizes exceeding 15 participants per group. Mixed-ANOVA revealed a significant reduction in CGI-S scores from T0 and T1 (F(1,33) = 13.703, *p* =0.001, η^2^ = 0.293). Additionally, a significant Group × Time interaction was also observed (F(1,33) = 5.233, *p* = 0.029, η^2^ = 0.137), showing that the reduction of CGI-S scores over time differed between the two groups. Post hoc analyses confirmed a significant decrease in CGI-S scores within the Li group (F(1,16) = 24.511, *p* < 0.001, η^2^ = 0.605), whereas no significant change was observed in the Control group (F(1,17) = 0.810, *p* = 0.381, η^2^ = 0.045).

With regard to C-GAS scores, mixed-design ANOVA showed a significant improvement from T0 to T1 (F(1,33) = 43.120, *p* < 0.001, η^2^ = 0.566), while no differential changes over time were observed between the two groups.

Extending the analysis to T2, results of the Mauchly’s Test did not show a violation of sphericity for Time factor for CGI-S (*p* = 0.460), whereas C-GAS showed a violation of sphericity (*p* = 0.043), so we used the Greenhouse–Geisser correction. A mixed-design ANOVA revealed a significant main effect of time for both outcome measures: a reduction in CGI-S scores (F(1,25) = 8.065, *p* = 0.009, η^2^ = 0.244) and an increase in C-GAS scores (F(1,25) = 16.196, *p* < 0.001, η^2^ = 0.393). However, no Group × Time interaction was found, suggesting that both groups improved similarly over time.

Adverse effects recorded at T1 fell into six categories: metabolic (38%, weight gain), neurological (27%, mainly sedation, occasionally motor disturbances), endocrinological (22%, mostly thyroid dysfunction), renal (8%), gastroenterological (3%), and dermatological (3%). No cardiac or hematological adverse events were observed. Renal, dermatological, and endocrinological side effects occurred only in the Li group. At T2, no new adverse events were reported; however, previously identified metabolic and endocrinological adverse events persisted in several cases.

## 4. Discussion

The present retrospective study aimed to evaluate the impact of adjunctive lithium therapy on symptom severity, global functioning, and tolerability profile in a sample of adolescents presenting with severe emotional dysregulation, independent of their specific diagnostic classification.

Descriptive analysis of the total sample revealed a clear predominance of female participants. This finding may reflect the overrepresentation of borderline personality organization, a condition significantly more frequent among females, as consistently reported in the literature [32,33].

Most of the baseline clinical characteristics, including DSM-5-TR categorical diagnoses [10], and the presence of self-harming or suicidal behaviors, did not significantly differ between the two groups. This suggests that the examined variables are unlikely to have acted as confounding factors in the interpretation of outcome measures. Moreover, the distribution of mean DERS subscale scores was consistent with previous findings, with higher scores typically observed in the “Strategies”, “Goals”, and “Impulse” dimensions, reflecting the core domains of emotional dysregulation commonly reported in adolescent populations [34]. Among the DERS dimensions, a significant group effect was found only for the Awareness subscale, with higher scores observed in the Li group. This finding suggests that adolescents receiving lithium may have greater difficulty in recognizing their emotional states, a feature that could reflect a characteristic of more severe clinical presentations requiring lithium treatment.

Other statistically significant baseline differences between the two groups involved the number of previous hospitalizations and the history of prior pharmacological treatments, both of which were more prevalent in the Li group. This finding is likely attributable to the longer illness duration observed in this subgroup, compared to the Control group. These data suggest that lithium was typically introduced as a later-stage intervention, following the partial or complete failure of previous treatment attempts. This pattern is consistent with the existing literature, which describes lithium as a second-line or adjunctive therapy in the treatment of complex or refractory cases, rather than a first-line option [35].

In the total sample, the most frequently prescribed medications were second-generation antipsychotics, followed by serotonergic agents and, to a lesser extent, antiepileptics. Notably, the latter were less commonly used in the Li group, likely due to the role of lithium as the principal mood stabilizers in these patients. This pharmacological distribution is partially consistent with general trends reported in the literature on psychotropic prescriptions in adolescents, although most available data are not stratified by specific diagnostic categories. In the Italian context, antidepressants and antipsychotics represent the most commonly prescribed psychotropic medications in pediatric populations [36]. However, epidemiological data on the use of mood stabilizers, particularly lithium, during adolescence remain limited [37].

An Italian study involving 30 adolescents diagnosed with bipolar disorder reported that, in addition to lithium, 57% of patients received antipsychotics, 13% antiepileptics, and 7% antidepressants [38]. A larger study in adults with borderline personality disorder found that 70% were prescribed antipsychotics and antidepressants, 33% antiepileptics, and only 4% lithium [39]. These data highlight the relatively limited use of lithium in clinical populations other than bipolar disorder, despite its potential therapeutic benefits.

Within the Li group, mean serum lithium levels were 0.75 mEq/L (SD = 0.17) at T1 and 0.72 mEq/L (SD = 0.17) at T2, placing them at the lower end of the therapeutic range reported in the literature, which typically spans from 0.79 to 1.2 mEq/L [19]. It is important to note, however, that these reference values are primarily drawn from studies focusing on specific diagnostic categories. In particular, mean serum lithium levels tend to be higher in patients with bipolar spectrum disorders compared to those with depressive or conduct disorders, closer to the values observed in our sample.

In terms of primary clinical outcomes, both groups experienced a reduction in symptom severity at 6 months, with the lithium group exhibiting greater improvement. At 12 months, symptom severity remained reduced in both groups, and the differences between them were no longer significant. Similarly, global functioning improved progressively in both groups across time points, reflecting overall functional enhancement over the course of treatment.

Evidence from the literature supports the use of lithium in combination with other psychotropic agents in pediatric populations. In adolescents with bipolar disorder, lithium–antipsychotic combinations have shown comparable efficacy and tolerability to valproate–antipsychotic regimens [40], and lithium combined with valproate has been associated with superior clinical improvement compared to monotherapy [41], based on outcome measures similar to those employed in the current study.

Although lithium is recommended by international guidelines as a second-line or adjunctive treatment for emotional dysregulation in individuals with borderline personality disorder, most studies focused on its impact on impulsivity and behavioral dyscontrol [42]. In our study, secondary outcomes revealed that the Li group showed a significantly higher mean number of prescribed psychotropic medications at 6 months and a greater number of psychiatric hospitalizations at the 12-month follow-up compared to the Control group. These findings may reflect the greater clinical complexity or treatment resistance typically characterizing patients for whom lithium therapy is indicated. The increased medication prescription observed at 6 months could be associated with the need to manage more severe or refractory symptomatology. Moreover, lithium requires a careful and often prolonged titration phase to achieve and maintain therapeutic serum levels. During this period, clinicians may need to introduce or maintain additional medications to ensure clinical stability. Supporting this interpretation, the difference in medication load between the groups appeared to decrease at 12 months, potentially indicating that once therapeutic lithium levels were stabilized, the need for adjunctive medications diminished. However, despite comparable reductions in clinical severity across groups, the greater number of hospitalizations observed in the Li group at 12 months may still reflect the underlying clinical complexity and higher vulnerability to acute decompensations in this subgroup. Other secondary measures, such as adverse effects and treatment adherence, remained comparable across groups, suggesting that lithium was generally well tolerated and did not negatively impact compliance.

Metabolic (weight gain) and neurological (mainly sedation) adverse effects were the most frequently reported in our sample. Notably, a distinct pattern of side effects emerged between the two groups: neurological adverse effects were more prevalent in the Control group, whereas endocrinological and renal side effects were almost exclusively reported in the Li group. Weight gain was observed at comparable rates across both groups. These findings are consistent with the existing literature on the side effect profile of lithium, particularly regarding its endocrine and renal impact [43]. They underscore the importance of regular monitoring of renal and thyroid parameters during lithium therapy, particularly in adolescent populations, where long-term exposure may carry developmental implications. The endocrine alterations observed in our sample, primarily subclinical hypothyroidism, were successfully managed with hormone replacement therapy and did not necessitate discontinuation of lithium treatment. In contrast, the greater prevalence of neurological side effects (e.g., extrapyramidal symptoms, tremors, dystonia, and parkinsonism) in the Control group may reflect the profile of alternative psychotropic agents, such as antipsychotics or anticonvulsants, which commonly impact the central nervous system [44].

These findings suggest that, although lithium requires careful clinical monitoring, its use in adolescents does not appear to compromise treatment adherence or substantially increase the overall burden of adverse effects.

Notably, no patient discontinued lithium therapy due to tolerability issues. This observation is in line with the limited but growing evidence on the safety of lithium in pediatric populations, which generally derives from studies with shorter follow-up durations (typically 8–14 weeks) and often excludes patients receiving polytherapy [24].

Considering the chronic and multifaceted nature of the disorders under investigation, extended follow-up periods are essential to capture the long-term efficacy and functional impact of treatment strategies. Supporting this need, a recent 10-year naturalistic longitudinal study in adolescents with bipolar disorder showed that lithium, used either alone or as part of combination therapy, was associated with better outcomes in targeted symptom domains and psychosocial functioning [45].

## 5. Conclusions

While these findings are preliminary due to the small sample size and should be interpreted with caution, they suggest that lithium salts, when administered as adjunctive therapy to other psychotropic medications, may be effective in reducing symptom severity and improving functional outcomes in adolescents with severe emotional dysregulation. Moreover, the addition of lithium did not negatively impact treatment tolerability or adherence. These results may encourage a reconsideration of prescribing attitudes toward lithium in youth. Historically, its use in individuals under 18 has been limited by concerns—often extrapolated from adult populations—regarding safety and tolerability, as well as its traditional association with bipolar disorder. Furthermore, the strong association between lithium and bipolar disorder in adult psychiatry has been uncritically extended to adolescents, despite important differences in clinical presentation and the increasing recognition of bipolar spectrum conditions in pediatric populations [46].

Although focusing on a specific dimension rather than categorical diagnoses may limit direct comparisons with the existing literature—largely based on diagnostic criteria—it offers a more accurate reflection of contemporary clinical practice. Adolescents with severe emotional dysregulation constitute a growing and clinically significant subgroup within child and adolescent mental health services. Overlapping symptoms such as suicidal and parasuicidal behaviors, mood instability, and conduct disturbances are difficult to classify using traditional nosological systems. Diagnostic inconsistency across centers and poor responsiveness to standard treatments are common, with non-adherence frequently intrinsic to the nature of the disorder. Therefore, this study represents a preliminary step towards the development of prospective research evaluating the medium- and long-term effects of lithium therapy in adolescents with severe emotion dysregulation. Given growing evidence of lithium’s anti-suicidal effects in adults, its potential in high-risk adolescent populations needs further investigation.

On the other hand, this study presents several limitations, most notably the relatively small sample size and the absence of a dedicated outcome measure specifically targeting emotional dysregulation as a distinct clinical dimension. Increasing the sample size would enable stratification by clinical characteristics, symptom profiles, and pharmacological regimens, thereby allowing for more accurate comparisons of the efficacy and safety of lithium as an adjunctive treatment relative to other psychopharmacological combinations. As with all retrospective studies, the present analysis is subject to potential selection and information bias. Although we attempted to reduce these risks by applying consistent inclusion criteria and extracting data systematically, the reliance on medical records may still limit the completeness and standardization of the data collected. Additionally, adverse events were classified by frequency rather than clinical severity, limiting the ability to distinguish between mild, moderate, and severe side effects. Finally, given the observational nature of the study, caution is warranted in interpreting the findings as causal. Prospective or randomized trials are needed to establish a stronger causal relationship between lithium use and clinical outcomes.

## Figures and Tables

**Table 1:** Demographic and clinical variables at T0.

	Control Group (*n* = 18)	Li Group (*n* = 17)	F	*p*-Value	η^2^
Age in years, mean (SD)	14.72 (1.23)	15.76 (1.82)	3.986	0.054	0.108
Number of psychotropic medications, mean (SD)	1.22 (0.42)	2.11 (0.70)	21.270	<0.001 *	0.392
Time from symptom onset to drug initiation in months, mean (SD)	17.89 (13.57)	15.12 (10.60)	0.450	0.507	0.013
Number of previous hospitalizations, mean (SD)	0.67 (0.77)	2.53 (1.28)	27.628	<0.001 *	0.456
CGI-S, mean (SD)	4.44 (0.51)	4.71 (0.47)	2.473	0.125	0.070
C-GAS, mean (SD)	42.22 (4.58)	40.53 (4.17)	1.301	0.262	0.038
	**Control group** **(*n* = 18)**	**Li group** **(*n* =17)**	**χ^2^**	***p*-value**	**Cramér’s V**
Gender (F/M ratio)	16/2	15/2	0.004	0.952	0.010
Family history of psychiatric disorder, yes/no (percentage)	8/10 (44.4%)	10/4 (71.4%)	5.770	0.056	0.406
Lifetime suicidal attempt(s), yes/no (percentage)	10/8 (55.5%)	10/7 (58.8%)	0.038	0.845	0.033
Lifetime self-injurious behavior, yes/no (percentage)	15/3 (83.3%)	16/1 (94.1%)	1.005	0.316	0.169
Prior psychotherapy, yes/no (percentage)	8/10 (44.4%)	10/7 (58.8%)	2.380	0.304	0.261
Prior pharmacotherapy, yes/no (percentage)	4/14 (22.2%)	17/0 (100%)	22.037	<0.001 *	0.793

* *p* < 0.05. SD = standard deviation. CGI-S = Clinical Global Impression–Severity scale; C-GAS = Children’s Global Assessment Scale.

**Table 2:** DERS scores at T0.

DERS Scores, Mean (SD)	Control Group (*n* = 18)	Li Group (*n* = 17)	F	*p*-Value	η^2^
Awareness	16.78 (4.81)	20.82 (6.39)	4.508	0.041 *	0.120
Clarity	18.50 (4.57)	19.24 (5.07)	0.204	0.655	0.006
Non-acceptance	18.56 (6.93)	20.82 (6.61)	0.980	0.329	0.029
Strategies	31.33 (7.66)	31.94 (7.04)	0.060	0.809	0.002
Impulse	22.89 (7.06)	22.76 (5.62)	0.003	0.955	0.000
Goals	21.61 (3.55)	21.12 (4.03)	0.148	0.703	0.004

* *p* < 0.05. SD = standard deviation; DERS = Difficulties in Emotion Regulation Scale.

**Table 3:** Between-group comparisons of clinical and treatment outcomes at T1 and T2.

T1		Control Group (*n* = 18)	Li Group (*n* = 17)	F	*p*-Value	η^2^
Number of psychiatric hospitalizations, mean (SD)		0.28 (0.75)	0.12 (0.33)	0.650	0.426	0.019
Psychotropic medications, mean (SD)		1.44 (0.70)	2.23 (0.66)	11.760	0.002 *	0.261
				**χ^2^**	***p*-value**	**Cramér’s V**
Side effects, percentage	0	33.3%	11.8%	2.974	0.226	0.291
	1	50%	52.9%			
	>1	16.7%	35.3%			
Treatment adherence, percentage	Good	50%	47%	1.031	0.597	0.172
	Moderate	27.8%	41.2%			
	Low	22.2%	11.8%			
**T2**		**Control group** **(*n* = 15)**	**Li group** **(*n* =12)**	**F**	***p*-value**	**η^2^**
Number of psychiatric hospitalizations, mean (SD)		0.20 (0.41)	0.75 (0.75)	5.829	0.023 *	0.189
Psychotropic medications, mean (SD)		1.60 (0.91)	2.17 (0.94)	2.517	0.125	0.091
				**χ^2^**	***p*-value**	**Cramér’s V**
Side effects, percentage	0	40%	25%	1.747	0.417	0.254
	1	60%	66.7%			
	>1	0%	8.3%			
Treatment adherence, percentage	Good	53.3%	50%	1.929	0.381	0.267
	Moderate	33.3%	16.7%			
	Low	13.3%	33.3%			

* *p* < 0.05. SD = standard deviation.

**Table 4:** Mixed-design ANOVA for symptom severity and global functioning.

T0–T1				
Variable	Effect	F	*p*-Value	η^2^
CGI-S	Time	13.703	0.001 *	0.293
	Group × Time	5.233	0.029 *	0.137
	Group	0.003	0.954	0.000
C-GAS	Time	43.120	<0.001	0.566
	Group × Time	1.374	0.250	0.040
	Group	0.153	0.699	0.005
**T0–T2**				
**Variable**	**Effect**	**F**	***p*-value**	**η^2^**
CGI-S	Time	8.065	0.009 *	0.244
	Group × Time	0.122	0.730	0.005
	Group	0.629	0.435	0.025
C-GAS	Time	16.196	<0.001	0.393
	Group × Time	0.553	0.464	0.022
	Group	1.442	0.241	0.055

* *p* < 0.05. SD = standard deviation. CGI-S = Clinical Global Impression–Severity scale; C-GAS = Children’s Global Assessment Scale.

## Data Availability

The original contributions presented in the study are included in the article; further inquiries can be directed to the corresponding authors. The further data are not publicly available due to privacy.
